# Multivalent DNA Vaccines as a Strategy to Combat Multiple Concurrent Epidemics: Mosquito-Borne and Hemorrhagic Fever Viruses

**DOI:** 10.3390/v13030382

**Published:** 2021-02-27

**Authors:** Jingjing Jiang, Stephanie J. Ramos, Preeti Bangalore, Dustin Elwood, Kathleen A. Cashman, Sagar B. Kudchodkar, Katherine Schultheis, Holly Pugh, Jewell Walters, Jared Tur, Jian Yan, Ami Patel, Kar Muthumani, Connie S. Schmaljohn, David B. Weiner, Laurent M. Humeau, Kate E. Broderick

**Affiliations:** 1 Inovio Pharmaceuticals Inc., Plymouth Meeting, PA 19462, USA; jiangjingjing@gmail.com (J.J.); stephanie.ramos@inovio.com (S.J.R.); bangalorep@gmail.com (P.B.); Dustin.Elwood@inovio.com (D.E.); Katherine.Schultheis@inovio.com (K.S.); hollypugh22@gmail.com (H.P.); Jewell.Walters@inovio.com (J.W.); jared.tur@inovio.com (J.T.); jyanshao@gmail.com (J.Y.); Kate.Broderick@inovio.com (K.E.B.); 2 United States Army Medical Research Institute of Infectious Diseases, Fort Detrick, MD 21702, USA; kathleen.a.cashman.ctr@mail.mil (K.A.C.); apatel@wistar.org (A.P.); connie.schmaljohn@nih.gov (C.S.S.); 3 Vaccine & Immunotherapy Center, The Wistar Institute of Anatomy and Biology, Philadelphia, PA 19104, USA; skudchodkar@Wistar.org (S.B.K.); kmuthumani@gmail.com (K.M.); dweiner@Wistar.org (D.B.W.)

**Keywords:** DNA vaccine, multivalent vaccine platform, immunogenicity, in vivo electroporation, Ebola, Lassa, Dengue, Marburg, Zika, Chikungunya

## Abstract

The emergence of multiple concurrent infectious diseases localized in the world creates a complex burden on global public health systems. Outbreaks of Ebola, Lassa, and Marburg viruses in overlapping regions of central and West Africa and the co-circulation of Zika, Dengue, and Chikungunya viruses in areas with A. aegypti mosquitos highlight the need for a rapidly deployable, safe, and versatile vaccine platform readily available to respond. The DNA vaccine platform stands out as such an application. Here, we present proof-of-concept studies from mice, guinea pigs, and non-human primates for two multivalent DNA vaccines delivered using in vivo electroporation (EP) targeting mosquito-borne (MMBV) and hemorrhagic fever (MHFV) viruses. Immunization with MMBV or MHFV vaccines via intradermal EP delivery generated robust cellular and humoral immune responses against all target viral antigens in all species. MMBV vaccine generated antigen-specific binding antibodies and IFNγ-secreting lymphocytes detected in NHPs up to six months post final immunization, suggesting induction of long-term immune memory. Serum from MHFV vaccinated NHPs demonstrated neutralizing activity in Ebola, Lassa, and Marburg pseudovirus assays indicating the potential to offer protection. Together, these data strongly support and demonstrate the versatility of DNA vaccines as a multivalent vaccine development platform for emerging infectious diseases.

## 1. Introduction

Emerging infectious disease outbreaks have significantly increased in the past decades largely due to climate and environmental change, increased international travel and trade, and rapid population growth. Studies have shown that more than half of emerging infectious diseases originate from wildlife in areas with socio-economic disadvantages and limited infrastructure to control these outbreaks [1]. Concurrent outbreaks of multiple emerging infectious diseases further complicate the problem of containment in lower income regions. Multiple hemorrhagic fever viruses have been reported to occur in overlapping regions of Africa. Of note, the recent 2013–2016 Ebola virus (EBOV) outbreak occurred in a Lassa virus (LASV) endemic region (Guinea, Liberia, and Sierra Leone) leading to 30,000 cases with more than 10,000 deaths [2]. This is in addition to the 5000 annual Lassa related deaths in the area as well as periodic Marburg outbreaks in the sub-Saharan region [3,4]. Meanwhile, the co-circulation of mosquito-borne viruses is a growing concern in regions of South America and Southeast Asia. The incidence of Zika (ZIKV), Dengue (DENV), and Chikungunya (CHIKV) virus co-circulation have increased in regions where A. aegypti mosquitos are present [5,6,7,8,9]. These examples highlight the need for a rapidly deployable solution for containment. Prophylactic vaccines are considered to be one of the most cost-effective prevention for infectious diseases. A safe, efficacious vaccine targeting multiple infectious viruses could be beneficial to populations at risk, greatly decreasing the chance of a pandemic, and reducing the public health burden. While there are multiple vaccines currently under development, most target individual diseases with recent investigations in multivariant vaccine development including Zika and Chikungunya. While promising, licensed vaccines are only available for Ebola and Dengue, and none that target several diseases simultaneously [10,11,12]. 

The next-generation non-live vaccine approach, DNA vaccines, are an advantageous platform for multivalent infectious disease vaccine development compared to other vaccine platforms, such as live-attenuated, subunit or viral vectored vaccines. These benefits include the absence of pre-existing or acquired vector immunity; relatively rapid, and low-cost manufacturing methods; stable multi-agent formulation capability; no need for cold-chain storage; a favorable safety profile; and the ability to generate both humoral and cellular immune responses. Historically DNA vaccines against infectious diseases and oncological disorders have advanced into early phase clinical trials with limited success. Advancements in design (highly optimized DNA encoded immunogens) and delivery (in vivo electroporation (EP)) have greatly improved the immunogenicity of DNA vaccines in large animals and human subjects [13,14,15]. Recently we demonstrated in a Phase 2b clinical trial that the human papillomavirus (HPV) DNA vaccine VGX-3100 delivered via intramuscular injection with EP (IM-EP) is capable of driving an effective, therapeutic immune response in which vaccinated patients with cervical intraepithelial neoplasia not only saw regression of their lesions but also cleared the disease-causing virus [16]. Additionally, we have previously reported on an in vivo intradermal electroporation (ID-EP) device for administering DNA vaccines to the skin and demonstrated plasmid gene expression and functional immune responses using this delivery method [17]. This device is currently being used in early clinical trials and has already demonstrated induction of potent immunity of DNA vaccines against HIV (NCT02431767), EBOV (NCT02464670) [18], and the first-in-human ZIKV vaccine (NCT02809443) [19]. Most recently ID delivery of a DNA vaccine for COVID-19 by the same ID-EP device (INO-4800) was developed showing both immunogenicity and protection in rodents and NHPs [20,21]. INO-4800 has successfully completed a Phase I clinical trial (NCT04336410) and entered Phase II/III (NCT04642638), demonstrating rapid roll-out and successful immunogenicity of the platform [22].

Several vaccine platforms have been used for bivalent or multivalent hemorrhagic fever vaccine development. For example, a bivalent VSV-based vaccine, a common approach for EBOV, has been used to target MARV in parallel [23]. A multivalent adenoviral vaccine targeting several EBOV and MARV strains demonstrated protective efficacy in non-human primates [24]. However, there are drawbacks to using a viral vectored-based vaccine, such as anti-vector immunity that may prevent subsequent vaccination against other pathogens using the same viral platform. By avoiding the risks associated with VSV-based vaccines, multivalent DNA vaccines delivered by EP have been shown to be effective in preclinical models. Multivalent vaccines targeting EBOV and MARV showed protection in mice, guinea pigs, and non-human primates [25,26,27]. A bivalent EBOV/MARV DNA vaccine delivered by needle-free jet injection has also been evaluated in Phase 1 clinical trials [28,29]. Although deemed safe and tolerable, immune responses were modest and variable, which may perform better with the inclusion of EP as has been seen recently with a ZIKV vaccine [30].

Currently, there are no approved vaccines for the prevention of ZIKV or CHIKV viral infections and the use of the only licensed vaccine for DENV (Dengvaxia) has recently been limited under a precautionary recommendation from the WHO due to safety concerns [31,32,33]. A diverse range of vaccines individually targeting these viruses are under various stages of clinical development, including viral (live attenuated, inactivated, and recombinant viruses), protein/subunit, DNA, and RNA platforms [34,35,36] but no with limited reports of any vaccines simultaneously targeting ZIKV, DENV, and CHIKV.

We have previously demonstrated preclinical immunogenicity and protective efficacy of individual DNA vaccines targeting EBOV and MARV either alone or in combination with LASV [25,27]. Our data also illustrated immunogenicity and protective efficacy of individual DNA vaccines targeting ZIKV [37,38] and CHIKV [39], and DNA vaccines developed for DENV serotypes 1–4 showed robust immunogenicity in mice, guinea pigs and non-human primates (unpublished data). Here, we present proof-of-concept studies in mice, guinea pigs, and non-human primates for multivalent hemorrhagic fever virus (MHFV) and mosquito-borne virus (MMBV) vaccines, which demonstrate the versatility of DNA as a multivalent vaccine development platform for emerging infectious diseases.

## 2. Materials and Methods

### 2.1. Plasmid Vaccine Constructions

Multivalent Hemorrhagic Fever Virus (MHFV) Vaccine: The MHFV vaccine was generated as a combination of DNA vaccines targeting EBOV, MARV, and LASV as described below. The pEBOV plasmid encodes full-length Ebola Zaire glycoprotein (GP) with the SynCon consensus strategy as previously described [27]. The pMARV plasmid encodes full-length Marburg 2005 Angola strain GP proteins as previously described [27]. EBOV and MARV plasmids were codon optimized and cloned into modified pVAX1 mammalian expression vectors. The pLASV plasmid encodes a codon-optimized LASV Josiah glycoprotein complex precursor (GPC) as previously described [40]. The following plasmids were generated for the purposes of in vivo antigen expression studies: pEBOV-FLAG plasmid encoding the same protein as pEBOV labeled with a C-terminal DYKDDDDK FLAG tag; pMARV-His plasmid encoding the same protein as pMARV labeled with a C-terminal 6-His tag; pLASV-Myc plasmid encoding the same protein as pLASV labeled with a C-terminal EQKLISEEDL Myc tag. 

Multivalent Mosquito-borne Virus (MMBV) Vaccine: The MMBV vaccine was generated as a combination of DNA vaccines targeting ZIKV, DENV serotypes 1–4 and CHIKV antigens as described below. The pZIKV plasmid DNA construct encodes a consensus full-length ZIKV precursor of membrane (prM) and envelope (E) proteins as previously described [38]. The pCHIKV DNA construct encodes consensus CHIKV E3, E2, and E1 envelope proteins as previously described [39]. To generate DENV serotype-specific prM and envelope consensus sequences, DENV serotypes 1–4 prM gene sequences were used; DENV serotypes 1–4 envelope sequences representing the sequences from various regions of the world were collected from GenBank to avoid sampling bias. The DENV serotype-specific prM and envelope consensus protein sequence was obtained after performing multiple alignments. The DENV serotype-specific consensus prM and envelope sequences were assembled together to construct the prME consensus sequences. A highly efficient leader sequence was fused in frame upstream of the start codon to facilitate the expression. Furthermore, in order to have a higher level of expression, the codon usage of this gene was adapted to the codon bias of Homo sapiens genes. In addition, RNA optimization was also performed: regions of very high (>80%) or very low (<30%) GC content and the cis-acting sequence motifs such as internal TATA boxes, chi-sites, and ribosomal entry sites were avoided. The genes encoding modified DENV1–4 prME proteins were synthesized and sequence verified by GeneArt (Thermo Fisher, Waltham, MA). The synthetic engineered DENV1–4 prME genes were 2094, 2094, 2088, and 2094 bp in length and were subcloned into the expression vector pGX0001 at the BamHI and NotI sites for further study. These constructs were named pDENV1, pDENV2, pDENV3, and pDENV4. 

### 2.2. Animals and Vaccinations

Female C57BL/6 mice (6–8 weeks) and female Hartley guinea pigs (8–10 weeks) weighing around 500–600 g were used in this study and were group housed with ***ad libitum*** access to food and water. Mixed male and female Rhesus macaques weighing 2.25–6.25 kg were individually housed and acclimated for 4 weeks before experimentation under standard conditions. All animals were housed at BioTox Sciences (San Diego, CA, USA) and all housing, handling, and treatment protocols were approved and handled according to the standards of the Institutional Animal Care and Use Committee.

Mouse immunizations: For MMBV vaccine studies, mice were injected intramuscularly followed by CELLECTRA^®^ IM-EP on days 0, 14, and 28 for a total of three immunizations each with a dose of 25 µg pZIKV, 100 µg of mixed pDENV1–4 plasmids (25 µg each plasmid), and 25 µg pCHIKV over 3 treatment sites per animal. Sera were collected on days 0, 14, 28, and 35 for ELISAs and splenocytes on day 35 for IFNγ ELISpot analyses.

Guinea pig treatments for tissue pDNA expression: Guinea pigs were shaved and depilated 1 day before treatment. pDNA plasmids were formulated as a cocktail of 0.33 mg/mL each of pEBOV-FLAG, pMARV-His, and p-LASV-Myc plasmids at final concentration of 1 mg/mL total pDNA. Guinea pigs were then injected with 50 μL of the pDNA plasmid cocktail intradermally by the Mantoux method immediately followed by EP. Treated skin samples were collected 24 hours post treatment for immunofluorescence analyses.

Guinea pig immunizations: For MHFV vaccine studies, guinea pigs were injected intradermally using the Mantoux method on the flank followed by CELLECTRA^®^ ID-EP on days 0, 21, and 42 for a total of three immunizations at a dose of 0.1 mg in 0.1 mL each of the pEBOV, pMARV, and pLASV plasmids for a total of 0.3 mg pDNA. Immunizations were delivered as either a cocktail of plasmids spread across 3 treatment sites, or each plasmid was delivered into individual sites for a total of 3 treatment sites within the same animal. Sera were collected on days 0, 21, 42, and 63 for ELISAs. For MMBV vaccine studies, guinea pigs were injected intradermally using the Mantoux method on the flank followed by CELLECTRA^®^ ID-EP on days 0, 21, and 42 for a total of three immunizations with a cocktail of 0.1 mg in 0.1 mL each of the pZIKV, pDENV1–4, and pCHIKV plasmids for a total of 0.6 mg pDNA spread across 6 treatment sites per animal per treatment.

NHP immunizations: For the MHFV vaccine study, rhesus macaques were injected intradermally using the Mantoux method over the quadricep muscle followed by CELLECTRA^®^ ID-EP on weeks 0, 4, and 8 for a total of three immunizations at a dose of 1 mg in 0.2 mL each of the pEBOV, pMARV, and pLASV plasmids for a total of 3 mg pDNA. Plasmids were delivered individually across 2 sites for each plasmid for a total of 6 treatment sites. NHPs then received a booster immunization of the same at week 25. Sera and BD Vacutainer CPT whole blood were collected on weeks 0, 2, 6, 10, 24 and 27 for ELISAs and IFN-γ ELISpot analyses. For the MMBV vaccine study, rhesus macaques were injected intradermally using the Mantoux method over the quadricep muscle followed by CELLECTRA^®^ ID-EP on weeks 0, 4, and 8 for a total of three immunizations at a dose of 1 mg in 0.1 mL each of the pZIKV, pDENV1–4, and pCHIKV plasmids for a total of 6 mg pDNA. Immunizations were delivered as either a cocktail of plasmids spread across 6 treatment sites, or each plasmid was delivered into individual sites for a total of 6 treatment sites. Plasmid dose and injection volume were consistent between cocktail and individual formulation groups. Sera and BD Vacutainer^®^ CPT whole blood were collected on weeks 0, 2, 6, 10, and month 6 for ELISAs and IFN-γ ELISpot analysis. 

### 2.3. Mouse Splenocyte Isolation

Briefly, spleens from mice were collected individually in 5 mL of RPMI1640 media supplemented with 10% FBS (R10), processed into single cell suspensions with a gentleMACS Dissociator (Miltenyi Biotec, Auburn, CA, USA), and then centrifuged at 1500 rpm for 10 min. Cell pellets were resuspended in 5 mL of ACK lysis buffer (Life Technologies, Carlsbad, CA, USA) for 5 min at room temperature, and PBS was then added to stop the reaction. The samples were again centrifuged at 1500 rpm for 10 min, cell pellets resuspended in R10, and then passed through a 45 μm nylon filter before use in an ELISpot assay.

### 2.4. Guinea Pig Skin Processing and Immunofluorescence

Skin biopsies were fixed in 4% paraformaldehyde (Sigma-Aldrich, St. Louis, MO, USA at 4 °C overnight. The next day, skin biopsies were buffered in a 30% sucrose solution (Sigma-Aldrich, St. Louis, MO, USA) and stored at 4 °C. For sectioning, biopsies were embedded in O.C.T. compound (Sakura, Tokyo, Japan) and sectioned at a thickness of 15 μm using an OTF cryostat (Bright Instruments, Cambridge, UK). Sections were stained with unconjugated primary goat anti-FLAG (QED Bioscience, San Diego, CA, USA), mouse anti-His (Abcam, Cambridge, UK), or rabbit anti-Myc (Abcam, Cambridge, UK) antibodies. Sections were then stained with donkey anti-goat AF488 (Abcam, Cambridge, UK), donkey anti-rabbit AF555 (Life technologies, Carlsbad, CA, USA), and goat anti-mouse AF647 (Life technologies, Carlsbad, CA, USA) conjugated secondary antibodies, respectively. An additional stain, Hoechst 33342 (Life Technologies, Carlsbad, CA), was used to visualize nuclei. The slides were then mounted with Fluoromount (eBioscience, San Diego, CA, USA) and viewed by fluorescence microscopy using an Olympus BX51 with a U-TV1X-2/U-CMAD 3 combo camera for photo acquisition (Olympus, Melville, NY, USA). MagnaFire software was used to acquire the images.

### 2.5. Enzyme-Linked Immunospot (ELISpot) Assays

To assess cellular IFNγ responses, mouse or NHP interferon (IFN)γ ELISpot assays were performed using commercial Mabtech IFNγ ELISpot kits (Mabtech, Sweden). Briefly, 96-well ELISpot plates pre-coated with capture antibody were blocked with R10 medium overnight at 4 °C. The following day, 200,000 mouse splenocytes or NHP PBMCs in R10 media were added to each well and incubated at 37 °C in 5% CO_2_ in the presence of peptide pools consisting of 15-mers overlapping by 9 amino acids and spanning the length of the ZIKV-prME, CHIKV E1, E2, E3, or DENV1, 2, 3, 4-prME proteins (for MMBV); or EBOV, MARV, or LASV glycoproteins (for MHFV); DMSO (negative control); and ConA (positive control for mouse) or PMA plus ionomycin (positive control for NHP). After 18–20 h, plates were washed and developed according to the manufacturer’s protocols, and IFNγ positive spots were counted using an automated ELISpot reader (CTL, Shaker Heights, OH, USA). Antigen-specific responses were determined by subtracting the number of spots in DMSO-treated samples from peptide-treated wells. Results are shown for individual animal spot-forming units (SFU)/10^6^ PBMCs obtained for triplicate wells. 

### 2.6. Enzyme-Linked Immunosorbent Assays (ELISAs)

ELISAs were performed to determine sera antibody binding titers. Nunc ELISA plates were coated with 1 µg/mL of recombinant ZIKV primary envelope (Meridian Life Science, Memphis TN), DENV serotypes 1, 2, 3 or 4 (Prospec, East Brusnwick, NJ, USA), CHIKV E2 protein (Immune Technology, New York, NY, USA), recombinant Ebola glycoprotein (Sino Biologicals, 40442-V08B1), Marburg glycoprotein (GenScript, Piscataway, NJ, USA), or Lassa Josiah strain glycoprotein complex (GenScript, Piscataway, NJ, USA) in DPBS overnight at 4 °C. Plates were washed three times and then blocked with 3% BSA DPBS with 0.05% Tween 20 for 2 h at 37 °C. Plates were then washed and incubated with serial dilutions of mouse, guinea pig, or NHP sera and incubated for 2 h at 37 °C. Plates were again washed and then incubated with HRP conjugated-species specific secondary antibodies and incubated for 1 h at 37 °C. After the final wash plates were developed using SureBlue TMB 1-Component peroxidase substrate as the substrate and the reaction stopped with TMB stop reagent (KPL, Milford, MA, USA). Plates were then read at 450 nm within 30 min using a SpectraMax Plus 384 Microplate Reader (Molecular Devices, Sunnyvale, CA, USA). 

### 2.7. Pseudovirus Neutralization Assay

Pseudovirus production: HIV-based EBOV, MARV or LASV pseudoviruses expressing a luciferase reporter gene were generated by co-transfection of 293T cells with pEBOV, pMARV, or pLASV plasmids and envelope-defective NL43R-E_LUC (NIH AIDS reagent program) using Lipofectamine 3000 (Life Technologies, cat. L3000075). The pseudovirus-containing culture supernatant was harvested 72 h post transfection and then centrifuged at 500× *g* for 5 min. Pseudovirus aliquots were stored at −80 °C until use in neutralization assays. 

Pseudovirus neutralization: Sera samples were heat inactivated for 30 min at 56 °C prior to testing. Serial dilutions of heat inactivated sera were mixed with equal volumes of pseudovirus in DMEM supplemented with 10% FBS and 1% penicillin-streptomycin (D10) and incubated 1 h at 37 °C, then added to 293T cells that were seeded one day prior in 96-well cell culture plates. Following 72 h of incubation in 5% CO_2_ at 37 °C, luciferase signal was quantified by Bright-Glo Luciferase Assay System (Promega, cat. E2650) according to manufacturer’s instructions and the luminescence (RLU) was read with Spectra Max HTS plate reader (BioTek, Winooski, VT, USA). Neutralization was measured by the reduction in luciferase signal in sera comparison to infection controls. For each animal, neutralization titer was the maximum sera dilution with significant RLU value over day 0 background determined by two-way ANOVA. Neutralization specificities were confirmed using monoclonal antibodies against LASV (clone 25.1C, Zalgen Labs, cat. Ab02510C), EBOV (clone KZ52, Absolute Antibody, Oxford, UK), and MARV (clone Mr78, Absolute Antibody, Oxford, UK).

### 2.8. Statistical Analysis

Data were presented as min. to max. with all data points. The statistical difference between individual and cocktail formulation groups was assessed using Mann Whitney test. Within each group, the statistical differences between pre-immunization and post-immunization were assessed using Kruskal-Wallis test with Dunn’s multiple comparisons test. * *p* < 0.05, ** *p* < 0.01, *** *p* < 0.005. 

## 3. Results

### 3.1. Co-Expression of Multivalent Hemorrhagic Fever Virus Vaccine Antigens in Guinea Pig Skin Following Intradermal Electroporation Delivery

**Figure 1:**
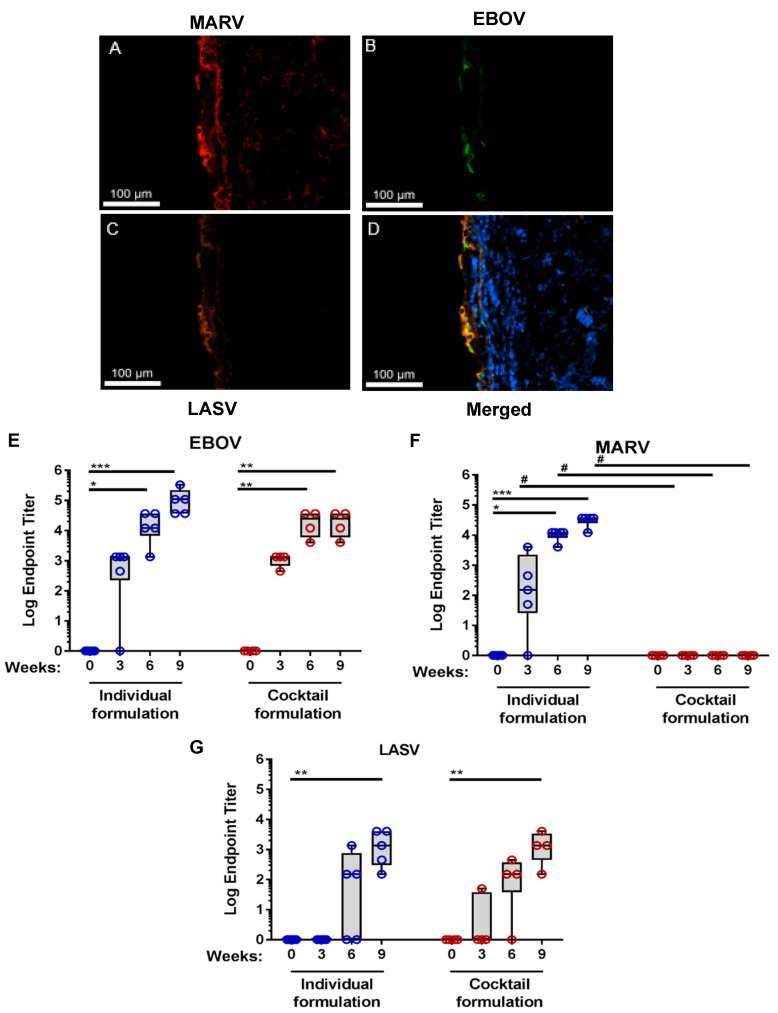
Co-expression of Ebola, Lassa, and Marburg glycoproteins in guinea pig skin and robust humoral response to MHFV DNA vaccine in guinea pigs. (**A**–**C**) Immunohistochemistry detection of encoded plasmid DNA antigens expressed in skin tissue 24 hours after delivery of co-formulated MHFV vaccine. Expression of plasmid-encoded MARV GP in red (**A**), EBOV GP in green (**B**), and LASV GP in yellow/orange (**C**) were detected by anti-His, Flag, and Myc tag antibodies respectively. Merged image illustrates co-localized signal for the majority of cells suggesting co-expression (**D**). (**E**–**G**) MHFV humoral response induced humoral response in guinea pigs. Hartley guinea pigs (*n* = 5/group) were immunized with a combination of pEBOV, pMARV, and pLASV plasmids as either individual plasmids (individual formulation, blue) or as a co-formulation (cocktail formulation, red) delivered by ID-EP on days 0, 21, and 42. Serum IgG antibody endpoint titers (EPTs) for individual guinea pigs were measured at the indicated timepoints by binding ELISA against EBOV GP (**E**), MARV GP (**F**), and LASV GP (**G**) antigens. The * *p* < 0.05, ** *p* < 0.01, *** *p* < 0.005 as compared with week 0 for the indicated timepoint; # is *p* < 0.05 comparing individual to cocktail formulation for the indicated timepoint.

Based on the success of other ID-EP administered DNA vaccines and adding to our multivalent DNA vaccine capabilities, we sought to investigate the possibility of ID-EP co-delivery of pEBOV, pMARV, and pLASV DNA vaccines as a multivalent hemorrhagic fever virus (MHFV) vaccine in Guinea pigs, a suitable small animal model of intradermal vaccine delivery [17]. In order to confirm in vivo expression of these three plasmids as a combined MHFV vaccine, guinea pigs were treated by ID-EP with a cocktail of pEBOV, pMARV, and pLASV plasmids and the skin collected 24 h later to measure antigen expression by immunofluorescent microscopy. All three glycoprotein (GP) antigens were expressed in the skin following ID-EP delivery (Figure 1A–C). Also, there were overlapping areas of EBOV, MARV, and LASV GP expression in treated skin, indicating co-transfection of tissue at the delivery site (Figure 1D). This data not only confirms successful delivery and expression of the MHFV DNA vaccine, but this level of visualization serves as a proof of concept for co-expression of multivalent DNA vaccines in the skin following intradermal delivery with CELLECTRA ID-EP. 

### 3.2. MHFV DNA Vaccine Immunogenicity in Guinea Pigs

Next, we sought to determine the cellular and humoral immunity generated by the MHFV DNA vaccine in the guinea pig model. Guinea pigs (*n* = 5 per group) received three ID-EP immunizations at three week intervals of 0.1 mg each of pEBOV, pMARV, and pLASV. Plasmids were either delivered as a cocktail formulation or separately into individual treatment sites on the same animal to assess the potential for plasmid interference. Humoral immune responses were measured by antigen binding IgG ELISA three weeks after each immunization. When plasmids were formulated individually, EBOV and MARV plasmids generated a robust and boost responsive humoral response, with 100% seroconversion after the second immunization, in guinea pigs (Figure 1E,F). Individually formulated LASV plasmid generated weaker antibody response (100% seroconversion after the third immunization, Figure 1G) compared with EBOV and MARV in guinea pigs. No cross reactivity was observed between pEBOV and pMARV induced antibodies (Supplement Figure S1). While the cocktail formulation of the MHFV vaccine showed 100% seroconversion after the first immunization for Ebola and 100% after the third immunization for Lassa, responses to Marburg suffered and individual formulation resulted in a better response to all three disease antigens (Figure 1E–G). 

### 3.3. MHFV DNA Vaccine Immunogenicity in NHPs

**Figure 2:**
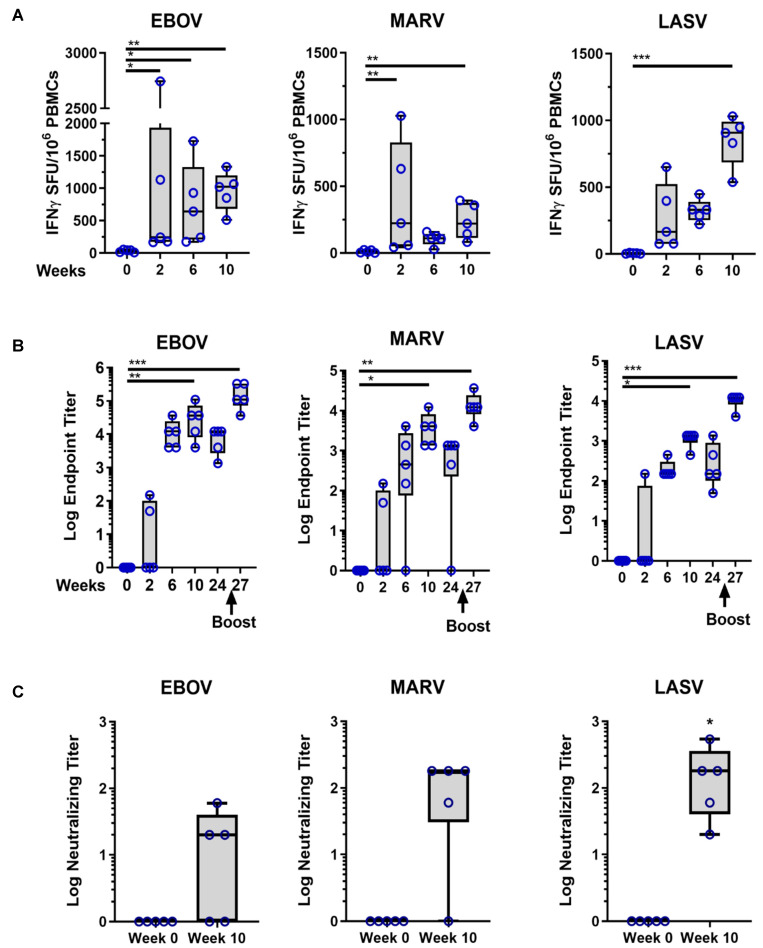
MHFV DNA Vaccine induces robust cellular and boostable humoral responses in Rhesus Macaques. Rhesus macaques (*n* = 5) were immunized with pEBOV, pMARV, and pLASV plasmids delivered by ID-EP as individual formulations on weeks 0, 4, and 8 and received a booster immunization at week 25. (**A**) Specific T cell responses for individual NHPs at the indicated timepoints were measured by IFNy ELISpot following stimulation of PBMCs with EBOV, MARV, or LASV GP peptide pools. Data represents SFUs (spot forming units) per million PBMCs for individual NHPs. (**B**) Serum IgG EPTs for individual NHPs at the indicated time points were measured by binding ELISA to EBOV, MARV, or LASV GP proteins. (**C**) Serum neutralizing antibody titers for individual NHPs at the indicated time points were measured by pseudovirus neutralization assays for EBOV, MARV, and LASV GP expressing pseudoviruses. Asterisks indicate a significant difference compared to Week 0 as described in the methods.

Based on results in guinea pigs, we then assessed individually formulated MHFV DNA vaccine immunogenicity using ID-EP delivery in the NHP model. Rhesus macaques (*n* = 5) were immunized with a total of 9 mg of multivalent HF plasmid DNA (3 mg each) ID with EP. Sera and PBMCs were collected at weeks 0, 6 and 10 to assess both cellular and humoral responses. The data in Figure 2A, shows that after three immunizations, multivalent immunizations induced strong antigen specific T cell responses against EBOV GP (955 ± 135, mean ± SE IFNγ SFU/million PBMCs), MARV GP (239 ± 60), and LASV GP (850 ± 85) antigens. 

Furthermore, the MHFV vaccines generated robust (100% seroconversion after the second immunization) and boostable humoral responses against EBOV GP and LASV GP, with a weaker response against MARV GP (80% seroconversion after second immunization and 100% seroconversion after third immunization) (Figure 2B). The antibody titers against all three GPs remained at high levels except for one NHP observed in the MARV-specific titer 16 weeks after the third immunization (Figure 2B). Even after receiving a boosting immunization at Week 25, the titers against all three antigens were enhanced as well. Protection from HF disease has been associated with neutralizing antibodies [41,42,43]. In order to evaluate the potential for protection with MHFV DNA vaccination, the neutralization ability of the NHP immune sera was tested by pseudovirus assay. Sera samples post the third immunization demonstrated neutralization titers from 20–60 for EBOV pseudovirus, from 60 to 180 for MARV pseudovirus, and from 20 to 540 for LASV pseudovirus (Figure 2C) exhibiting potential for protection from three three HF diseases using a DNA-based vaccine platform.

### 3.4. MMBV DNA Vaccine Immunogenicity in Mice

**Figure 3:**
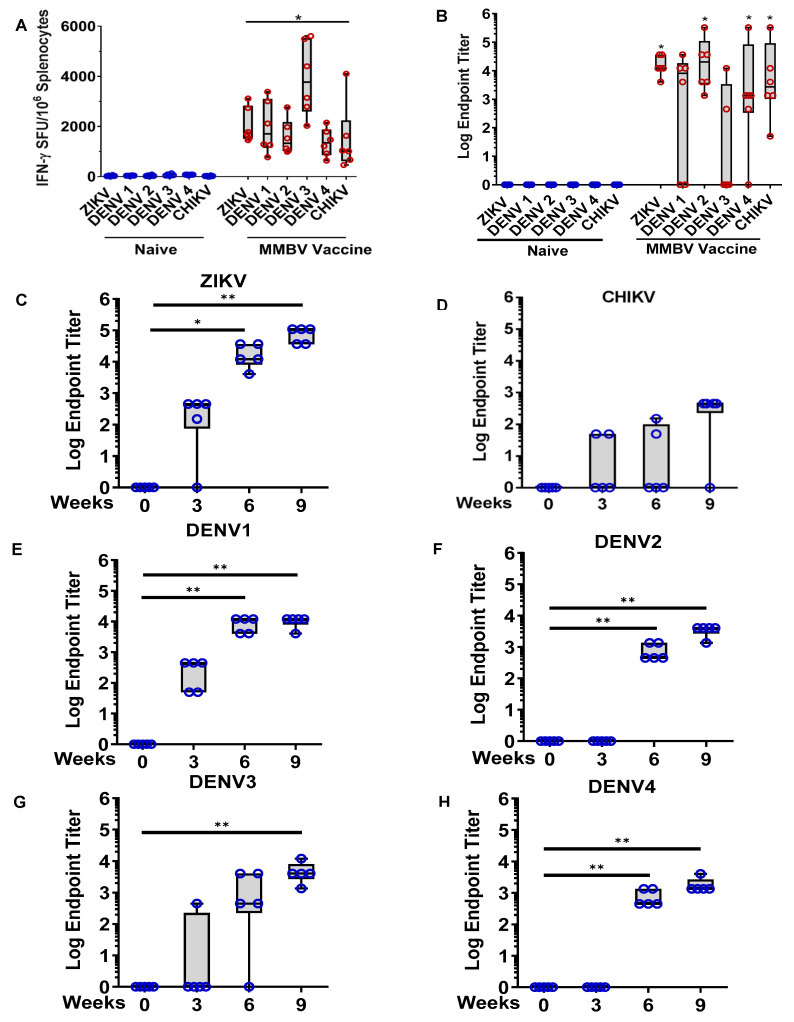
MMBV DNA vaccine induces cellular and humoral immune responses in mice and guinea pigs. (**A**,**B**) C57BL/6 mice (*n* = 6/group) were untreated (naïve) or immunized with MMBV DNA vaccine as a cocktail of pZIKV, pDENV1–4, and pCHIKV plasmids delivered by IM-EP on days 0, 14, and 28 and assessed for cellular (**A**) and humoral (**B**) immune responses two weeks post final immunization. (**A**) Isolated splenocytes were stimulated with the indicated viral envelope peptide pools and antigen-specific T cells detected by IFNγ ELISpot assay. Data represents SFUs per million splenocytes for individual mice. (**B**) IgG antibodies in serially diluted sera samples were measured by binding ELISA against the indicated viral envelope proteins. Data represents binding IgG EPTs for individual mice. Asterisks indicate a significant difference in IFNγ SFUs or IgG EPTs compared to naïve mice as described in the methods. (**C–H**) Hartley guinea pigs (*n* = 5) were immunized with MMBV DNA vaccine as a cocktail of pZIKV, pDENV1-4, and pCHIKV plasmids delivered by ID-EP on days 0, 21 and 42. Serum IgG antibody endpoint titers (EPTs) for individual guinea pigs were measured at the indicated timepoints by binding ELISA against ZIKV (**C**), CHIKV (**D**), DENV1 (**E**), DENV2 (**F**), DENV3 (**G**), and DENV4 (**H**) envelope proteins. Data represents binding IgG EPTs of individual guinea pigs. Asterisks indicate significant difference in IgG EPTs compared to Week 0 as described in the methods.

Immunogenicity and protective efficacy of individual pZIKV, pDENV1–4, and/or pCHIKV DNA vaccines were previously evaluated in mice and NHP models published [37,38,39,44] and unpublished data. Given the potential benefits of simultaneous vaccination against ZIKV, DENV, and CHIKV, we sought to determine if this could be done using a combination of these plasmids as a multivalent mosquito-borne virus (MMBV) DNA vaccine. We first assessed the immunogenicity of the MMBV vaccine in mice after three immunizations by IM-EP delivery of 25 µg each of pZIKV, pDENV1, pDENV2, pDENV3, pDENV4, and pCHIKV, individually (Supplement Figure S2) or as cocktail (Figure 3A,B). Cellular responses were measured by IFNγ ELISpot one week after the final immunization. As shown in Figure 3A, the MMBV vaccine induced a strong IFNγ response against ZIKV (2052 ± 285, mean ± SE SFU/10^6^ splenocytes), DENV1 (1973 ± 428), DENV2 (1569 ± 284), DENV3 (3911 ± 606), DENV4 (1367 ± 228), and CHIKV (1485 ± 5488) antigens. Sera collected from these mice were used to evaluate humoral responses by antigen binding IgG ELISAs against all six target antigens. The data in Figure 3B illustrate that MMBV vaccine induced strong binding antibodies with 100% seroconversion rates against ZIKV, DENV2, and CHIKV envelope proteins (binding endpoint titer [EPT] ranges of 4050 to 36,450, 1350 to 328,050 and 50 to 328,050, respectively). DENV1, 3, and 4 binding antibodies were also detected, but with lower seroconversion rates (4/6, 2/6, and 5/6 respectively) (Figure 3B). Together these data indicate that a combination of ZIKV, DENV, and CHIKV DNA vaccines is immunogenic in mice providing support for further testing in larger animal species.

### 3.5. MMBV DNA Vaccine Immunogenicity in Guinea Pigs

We next evaluated the immunogenicity of the MMBV DNA vaccine in guinea pigs. Guinea pigs received three immunizations spaced three weeks apart by ID-EP delivery of a cocktail of 0.1 mg each of pZIKV, pDENV1, pDENV2, pDENV3, pDENV4, and pCHIKV. Humoral immune responses were measured by antigen binding IgG ELISAs. MMBV DNA vaccination generated robust antibody responses against all six antigens, with 100% seroconversion against ZIKV and all four DENV serotypes, and 80% (4/5 animals) against CHIKV after completion of the full immunization regimen (Figure 3C–H). Cross reactive humoral responses with DENV1-4 could also bind to ZIKV antigen (Supplemental Figure S3). ZIKV, DENV1, DENV3 and CHIKV envelope-binding antibodies were detected in several guinea pigs after just one immunization (Figure 3C,D,E,G), and robust antibodies against ZIKV and DENV1–4 antigens were detected in all but one guinea pig after 2 immunizations (Figure 3C,E–H).

### 3.6. MMBV DNA Vaccine Immunogenicity in NHPs

**Figure 4:**
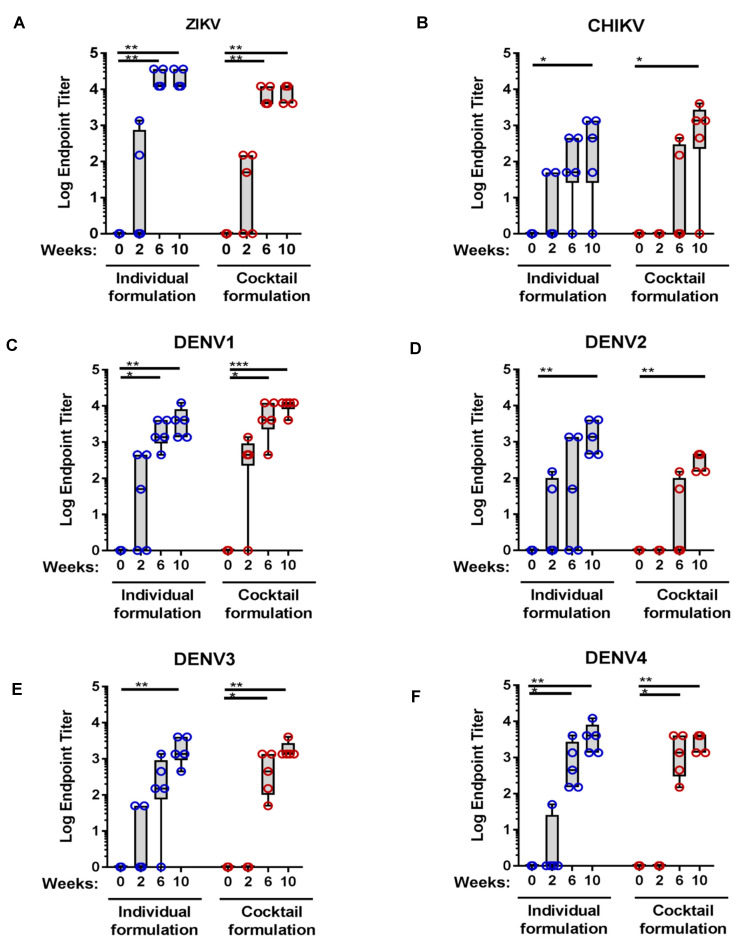
Humoral immune responses in NHPs after MMBV DNA vaccination via intradermal electroporation. Rhesus macaques (*n* = 5/group) were immunized with pZIKV, pDENV1–4, and pCHIKV plasmids delivered by ID-EP as either a cocktail formulation into the same treatment sites or individual formulation with distinct treatment sites for each plasmid. Serum IgG EPTs for individual NHPs at the indicated time points were measured by binding ELISA to ZIKV (**A**), CHIKV (**B**), DENV1 (**C**), DENV2 (**D**), DENV3 (**E**), and DENV4 (**F**) envelope proteins. Data represents binding IgG endpoint titers of individual NHPs. Asterisks indicate significant difference in IgG EPTs compared to Week 0 as described in the methods. There were no significant differences between individual and cocktail formulation treatment groups.

Following the positive results in mice and guinea pigs, we then assessed MMBV DNA vaccine immunogenicity using ID-EP delivery in the NHP model. Rhesus macaques (*n* = 5 per group) received three immunizations spaced four weeks apart by ID-EP delivery of 1 mg each of pZIKV, pDENV1, pDENV2, pDENV3, pDENV4, and pCHIKV. Plasmids were delivered either as a cocktail formulation or into individual treatment sites to assess the potential for plasmid interference. Humoral immune responses were measured by antigen binding IgG ELISA two weeks after each immunization. MMBV DNA vaccination generated robust, boostable humoral responses against all six antigens, with 100% seroconversion against ZIKV and all four DENV serotypes, and 80% (4/5 animals, each treatment group) against CHIKV after completion of the full immunization regimen (Figure 4). ZIKV, DENV1, DENV3, and DENV4 envelope-binding antibodies were detected in five out of five NHPs in the cocktail formulation group and in four out of five NHPs in the individual treatment group after two immunizations (Figure 4A,C,E,F). There were no significant differences in seroconversion rates or mean binding EPTs between the cocktail and individual formulation treatment groups for any of the six viral antigens at any time point tested.

**Figure 5:**
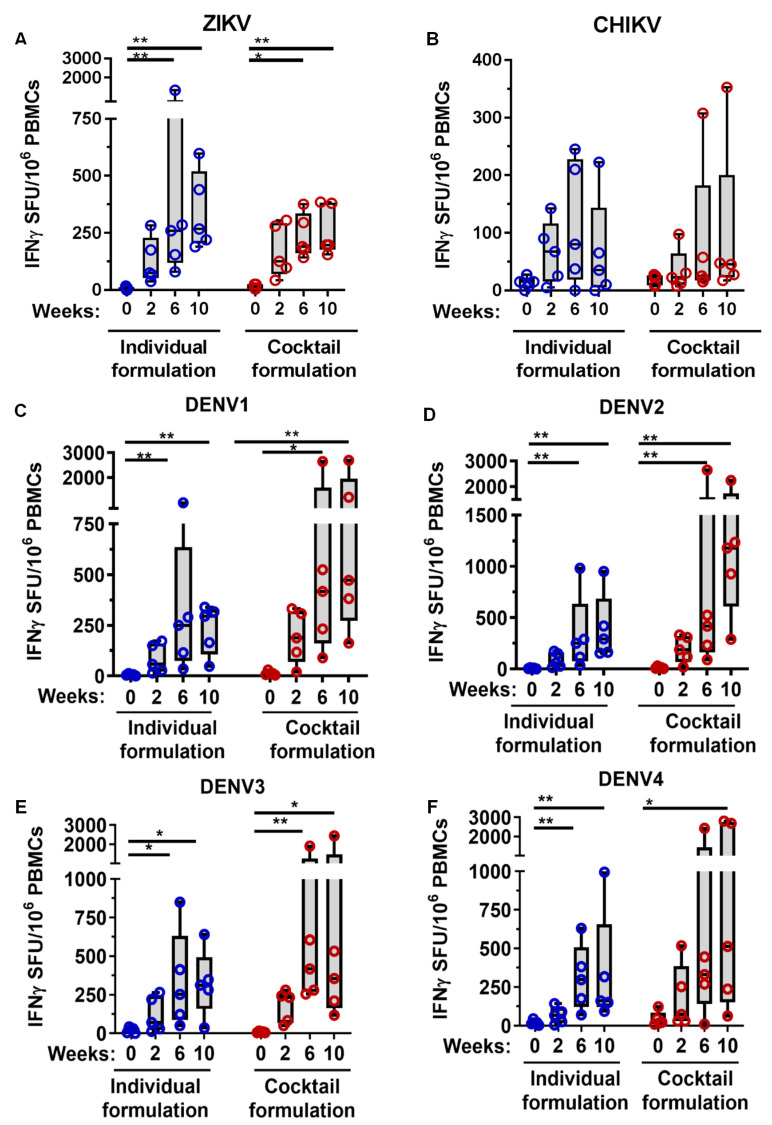
Cellular immune responses in NHPs after MMBV DNA vaccination via intradermal electroporation. Rhesus macaques were immunized as described in Figure 4. Specific T cell responses for individual NHPs at the indicated timepoints were measured by IFNy ELISpot following stimulation of PBMCs with (**A**) ZIKV, (**B**) CHIKV, (**C**) DENV1, (**D**) DENV2, (**E**) DENV3, and (**F**) DENV4 envelope peptide pools. The data represents SFUs per million PBMCs for each NHP. Asterisks indicate significant difference in SFUs compared to week 0 as described in the methods. There were no significant differences between individual and cocktail formulation treatment groups.

Cellular responses of MMBV DNA-vaccinated NHPs were measured by IFN-y ELISpot two weeks after each immunization (Figure 5). ID-EP delivery of both individual and cocktail formulations of MMBV DNA vaccine induced antigen specific T cell responses in all NHPs against ZIKV (343 ± 77 and 263 ± 49 mean ± SE IFNγ SFU/million PBMCs, respectively), DENV1 (233 ± 55 and 982 ± 462), DENV2 (397 ± 147 and 1175 ± 314), DENV3 (324 ± 96 and 730 ± 432 mean ± SE), and DENV4 (343 ± 167 and 1262 ± 611) envelope antigens after three immunizations (Figure 5A,C–F). CHIKV-specific T cell responses were lower than those against ZIKV and DENV for both individual and cocktail treatment groups (66 ± 41 and 98 ± 64 mean ± SE IFNγ SFU/million PBMCs, respectively) with 3/5 NHPs responding in the individual formulation group and 2/5 in the cocktail formulation group (Figure 5B). There were no significant differences in T cell responses between the cocktail and individual formulation treatment groups for any of the six viral antigens at any time point tested. Combined with the ELISA results, these data confirm that a multivalent DNA vaccine with ID-EP delivery can generate antibody and T cell responses to ZIKV, DENV serotypes 1–4, and CHIKV in NHPs.

**Figure 6:**
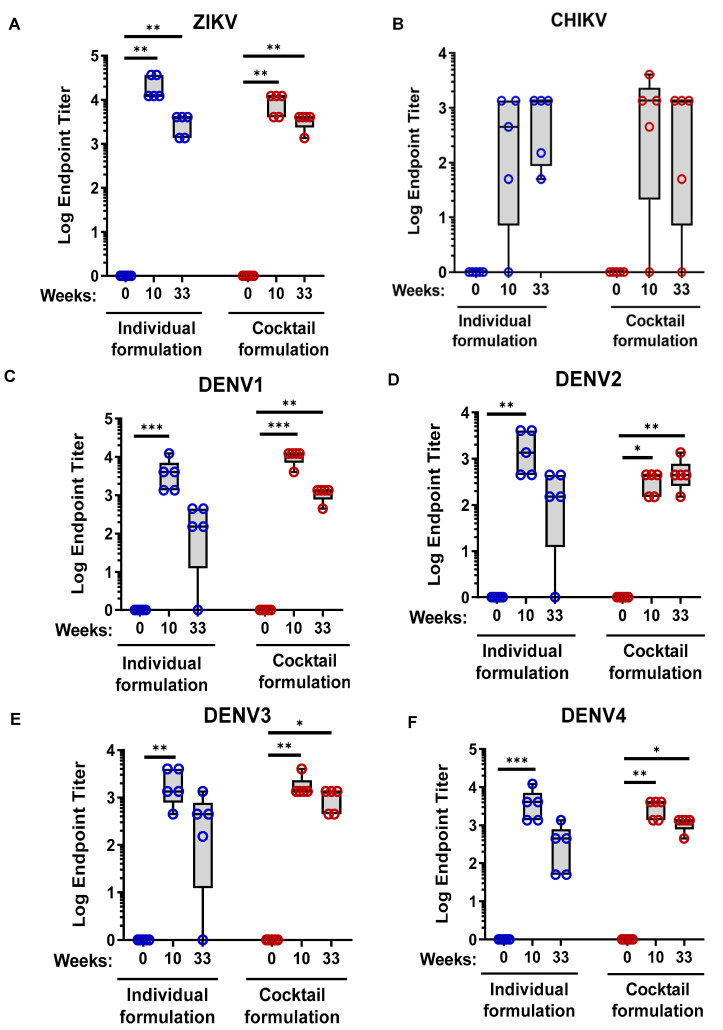
Durability of MMBV DNA vaccine induced humoral immune responses in NHPs. Rhesus macaques were immunized as described in Figure 4. Serum IgG EPTs for individual NHPs were measured at six months (week 33) post final immunization by binding ELISA to ZIKV (**A**), CHIKV (**B**), DENV1 (**C**), DENV2 (**D**), DENV3 (**E**), and DENV4 (**F**) envelope proteins. Data represents binding IgG endpoint titers of individual NHPs. The * *p* < 0.05, ** *p* < 0.01, *** *p* < 0.005. There were no significant differences between individual and cocktail formulation treatment groups.

We next sought to assess the longevity of MMBV DNA vaccine induced immune responses. Humoral and cellular responses of MMBV DNA vaccinated NHPs six months post final immunization (33 weeks post initiation of immunization) were measured by IgG binding ELISA and IFNγ ELISpot. Strong ZIKV, DENV1–4, and CHIKV binding antibody titers were detected up to six months post MMBV DNA immunization and were only modestly reduced compared to the 10-week time point (Figure 6A–F). Four out of five NHPs in the individual treatment group-maintained antibodies against all six target antigens with only one NHP losing DENV1, DENV2, and DENV3 binding antibodies. All 5/5 NHPs in the cocktail formulation group-maintained antibodies against all six target antigens excluding the one NHP that never generated CHIKV binding antibodies, yet still had robust ZIKV and DENV1-4 binding antibodies (Figure 6A–F). 

**Figure 7:**
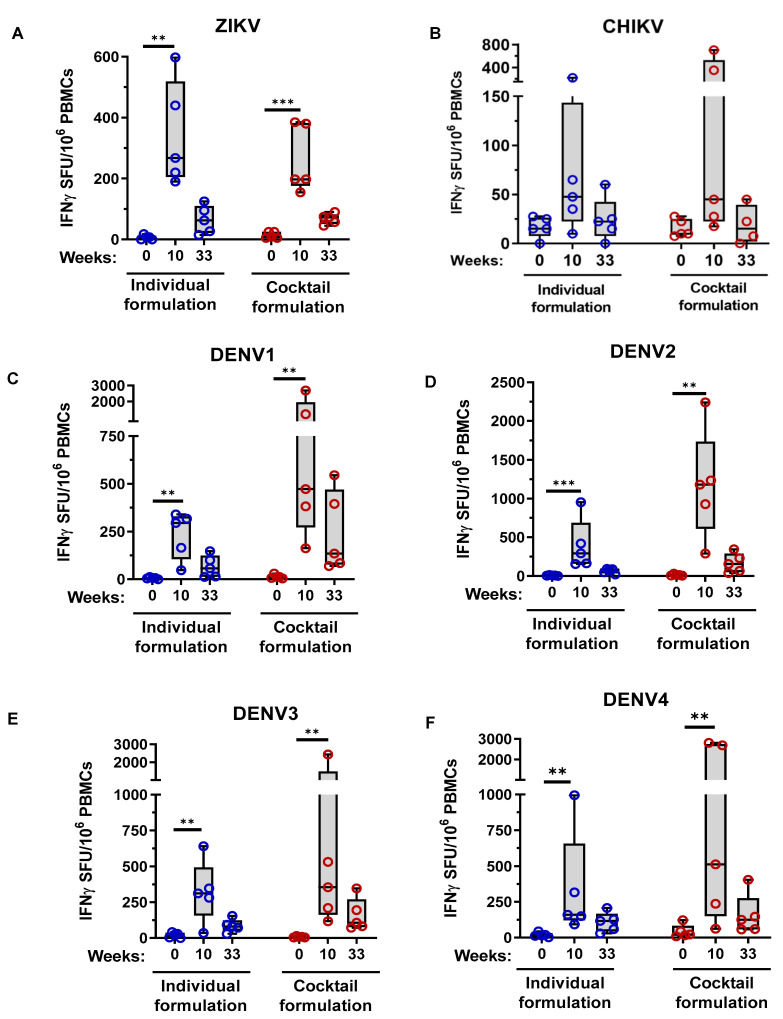
Durability of MMBV DNA vaccine induced cellular immune responses in NHPs. Rhesus macaques were immunized as described in Figure 4. Specific T cell responses for individual NHPs were measured at six months (week 33) post final immunization by IFNy ELISpot following stimulation of PBMCs with (**A**) ZIKV, (**B**) CHIKV, (**C**) DENV1, (**D**) DENV2, (**E**) DENV3, and (**F**) DENV4 envelope peptide pools. Asterisks indicate significant difference in SFUs compared to Week 0 as described in the methods. There were no significant differences between individual and cocktail formulation treatment groups.

As expected T cell responses were reduced six months post MMBV DNA immunization, but several NHPs from both treatment groups had detectable T cells against ZIKV (3/5 NHPs in the individual and 4/5 in the cocktail formulation group), DENV1 (3/5 and 5/5), DENV2 (3/5 and 4/5), DENV3 (4/5 and 5/5), and DENV4 (4/5 and 5/5) envelope antigens (Figure 7A,C–F). Of note, 2/5 individual and 3/5 cocktail MMBV DNA vaccinated NHPs had cellular responses against ZIKV and DENV1-4 antigens. No NHPs had detectable CHIKV-specific T cell responses six months post immunization (Figure 7B). There were no significant differences in mean binding antibody EPTs or ELISpot responses between the cocktail and individual formulation treatment groups for any of the six viral antigens at six months post immunization.

## 4. Discussion

Multiple concurrent emerging infectious viruses in endemic areas are a major threat to public health systems. One example mentioned earlier is the increase in reports about concurrent outbreaks of ZIKV, DENV, and CHIKV in A. aegypti mosquito prone areas. In fact, according to the WHO, more than 60 countries reported incidences of ZIKV infection during the 2015–2016 outbreak and over 100 countries are endemic for DENV and/or CHIKV [32,45,46]. In addition, the emergence of Ebola and Marburg viruses in West Africa, where the Lassa virus is already endemic has also stressed the necessity for a vaccine against multiple hemorrhagic fever viruses. The DNA vaccine platform stands out as a promising candidate for overcoming the obstacles associated with multiple HFV due to the advantages inherent in its manufacturing, storage, and improved safety profile. Requiring only DNA sequence information, these vaccines can be designed, manufactured, and delivered rapidly in response to imminent pathogenic threats, and can be stored at a wider temperature range compared with their viral vector counterparts. Inovio’s proprietary DNA formulation achieves DNA stability at room temperature for up to one year and greater than 2 years at 4 degrees [18,20], which is of particular importance in resource limited settings. 

Our study is one of the first reports of a multivalent vaccine generating a combined humoral and cellular immune response against ZIKV, DENV serotypes 1–4, and CHIKV in multiple preclinical models, including NHPs. MMBV DNA vaccine-induced immune responses were durable, lasting up to at least six months post immunization in NHPs. ZIKV and DENV specific antibody titers and IFNγ ELISpot responses in MMBV DNA vaccinated NHPs were comparable to those of other individual DNA vaccines reported as efficacious in NHP models of viral challenge [47,48,49]. Although MMBV DNA vaccine induced CHIKV immune responses were low in NHPs, they were within the range associated with protection [50]. Formulation options were compared as well, showing no statistical differences between individual and cocktail formulation. In cases such as this, a cocktail formulation is preferred to simplify both production and storage of the vaccine as well as administration to patients. Further preclinical studies are needed to evaluate the presence of neutralizing antibodies and protection from challenge, the potential for ADE antibody generation and to support clinical development of the MMBV DNA vaccine.

In this study, we also showed successful co-expression of the MARV, EBOV and LASV glycoproteins in guinea pig skin following skin delivery with the CELLECTRA 3P device, which led to 100% seroconversion for each of the three vaccine targets. We observed that immune responses to MARV were reduced when this MVHF vaccine was delivered as a cocktail in guinea pigs (Supplement Figure S1), possibly due to plasmid or immune interference [51]. This indicates that formulation evaluation is necessary for different multivalent vaccines during development. Based on our results, we decided to develop individual formulated MHFV vaccine in NHPs and are investigating optimizations, such as sequence optimizations or other plasmid formats, that may allow co-formulation without dampening immune responses to this antigen. Multiple factors could contribute to immunogenicity differences for multivalent vaccines, including plasmid interference, antigenic interference, differences in encoded antigen expression levels, and inherent differences in immunogenicity for different antigens. DNA plasmid interference can occur with specific plasmids but in many cases shows a reduction in responses to multiple vaccine antigens that are resolved by removing a single plasmid [52]. A recent study testing a multivalent vaccine that includes EBOV, SUDV, MARV, and LASV antigens showed no significant differences in immunogenicity between single and multivalent vaccines in NHPs suggesting that antigen/immune interference may not be the issue [53]. Interference was also not observed for a different vaccine combining EBOV and MARV antigens [24], although this vaccine used a different vector platform and a combination that did not include LASV. DNA combinations including EBOV, MARV with or without Sudan ebolavirus (SUDV) and Ravn virus antigens have shown some reduction in antibody production but no change in neutralizing antibodies or cellular responses. The reduction in antibodies was hypothesized to be due to a possible reduction in antigen expression as opposed to immune interference [26]. Although our analyses confirmed the expression of all encoded MVHF virus antigens at the treatment site, this methodology was not quantitative leaving differences in expression levels a possibility. These examples as well as our own internal studies show that reduction in response to a single antigen resultant from a cocktail formulation in rodent species is often not observed in larger animals such as NHPs. In the future we plan to pursue the optimization of the MARV DNA plasmid to allow for cocktail formulation through methods such as codon optimization, dose optimization, and other design refinements to restore the antigenicity of MARV.

Our results provide strong proof-of-concept in three animal species that a multivalent DNA vaccine delivered using minimally invasive ID-EP technology can induce combined cellular and humoral responses against multiple emerging viruses, which present a possible solution to the obstacle of a rapidly deployable multivalent vaccine for such critical diseases in resource limiting settings. Outbreaks similar to mosquito-borne and hemorrhagic fever diseases are difficult to predict and are likely to occur in the future with other targets. A multivalent strategy with a quick manufacturing response as presented here offers the potential for preventing existing and future threats including MERS-CoV, influenza, SARS-CoV, SARS-CoV-2/COVID-19, and future pandemics. 

## Data Availability

Not applicable.

## Supplementary Materials

The following are available online at https://www.mdpi.com/1999-4915/13/3/382/s1, Figure S1: Cross reactive antibody responses and plasmids interference among EBOV, MARV and LASV DNA vaccines in guinea pigs, Figure S2: Cross reactive cellular responses between DENV1-4, ZIKV and CHIKV vaccines in mice, Figure S3: Cross reactive humoral responses between DENV1-4, ZIKV and CHIKV vaccines in guinea pigs.
