# Supplementary material for: Multivalent DNA Vaccines as a Strategy to Combat Multiple Concurrent Epidemics: Mosquito-Borne and Hemorrhagic Fever Viruses
Source: Viruses. 2021 Feb 27;13(3):382. doi: 10.3390/v13030382 (PMC7997291; doi:10.3390/v13030382)
Supplement: Supplementary file 1 [file viruses-13-00382-s001.pdf]

Supplement Figure S1

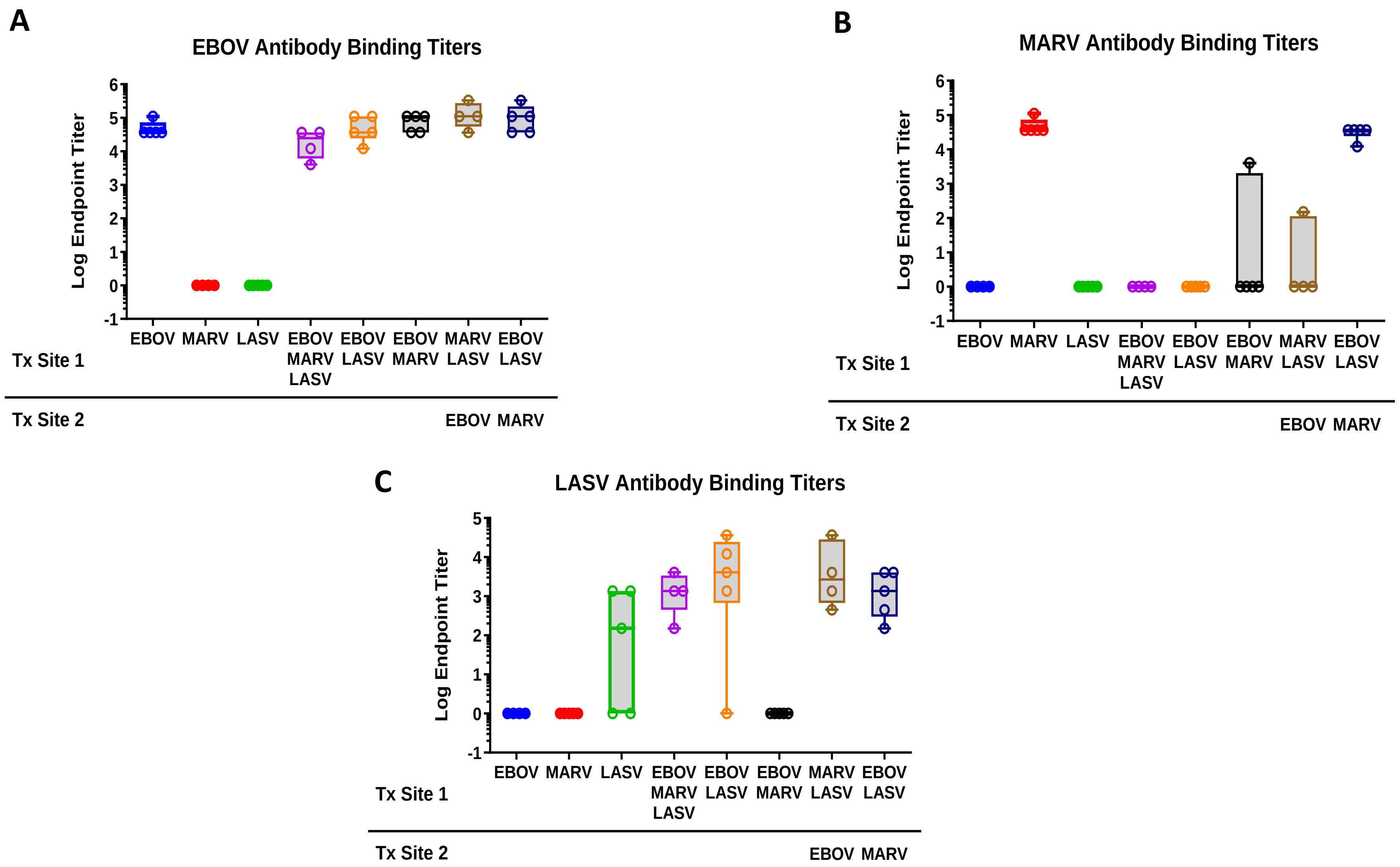

Supplemental Figure S1. Cross reactive antibody responses and plasmids interference among EBOV, MARV and LASV DNA vaccines in guinea pigs. Guinea pig sera were assessed at 9 weeks. EBOV-specific (A), MARV-specific (B) and LASV-specific (C) antibody end-point titers by ELISA showed that there is no cross reactivity between EBOV, MARV or LASV DNA vaccines and antibody responses to MARV was reduced when MARV vaccine was co-formulated with other vaccines.

Supplement Figure S2

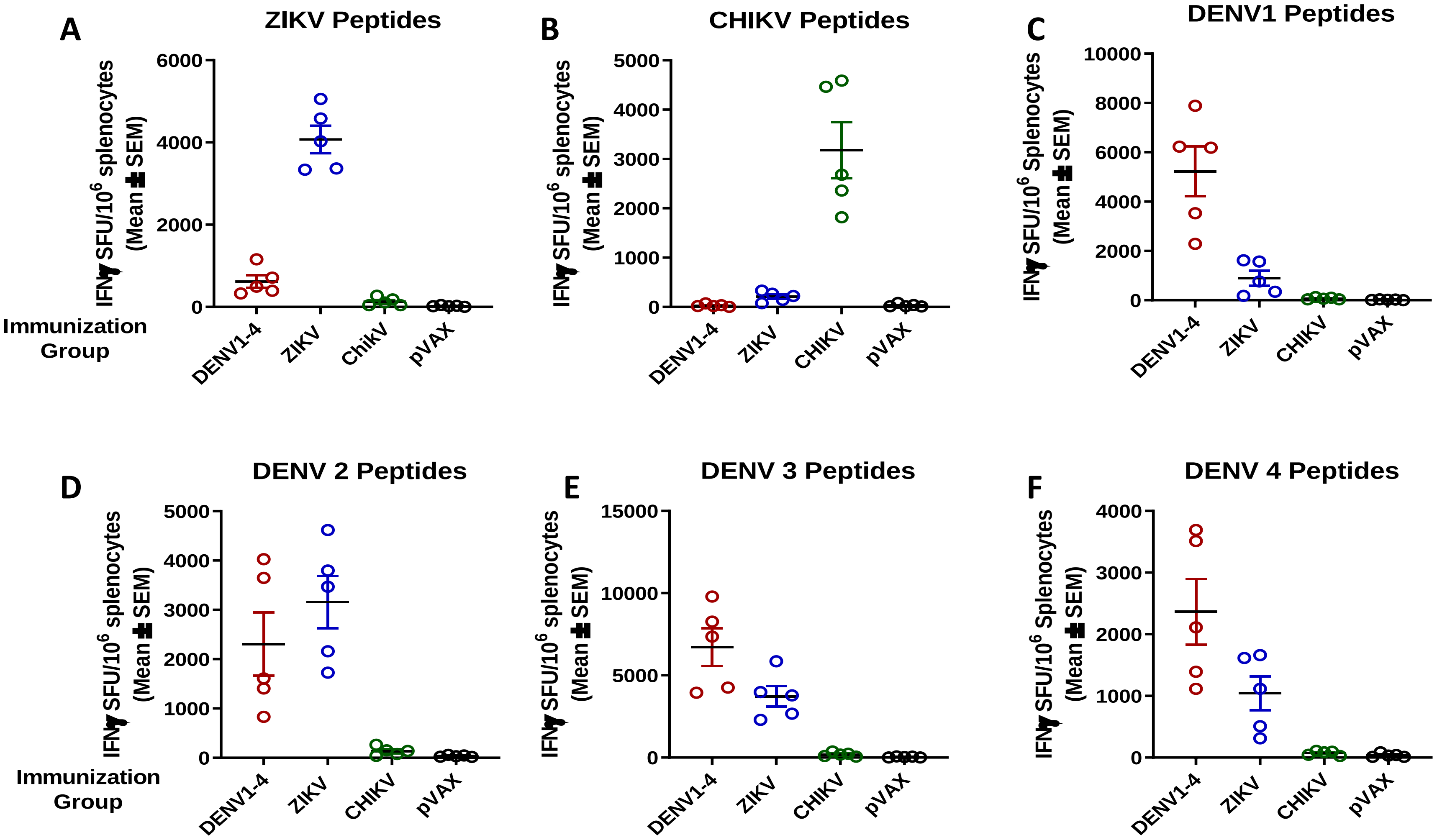

Supplement Figure S2. Cross reactive cellular responses between DENV1-4, ZIKV and CHIKV vaccines in mice. Mice were immunized with DENV1-4, ZIKV, CHIKV or pVAX (control). Splenocytes IFN-γ responses against ZIKV(A), CHIKV (B), DENV1-4 (C-F) peptides were measured. Cross reactive cellular response to DENV1-4 detected when animals immunized with ZIKV or DENV vaccine. No cross reactivity to DENV1-4 or ZIKV was detected with CHIKV immunization.

Supplement Figure S3

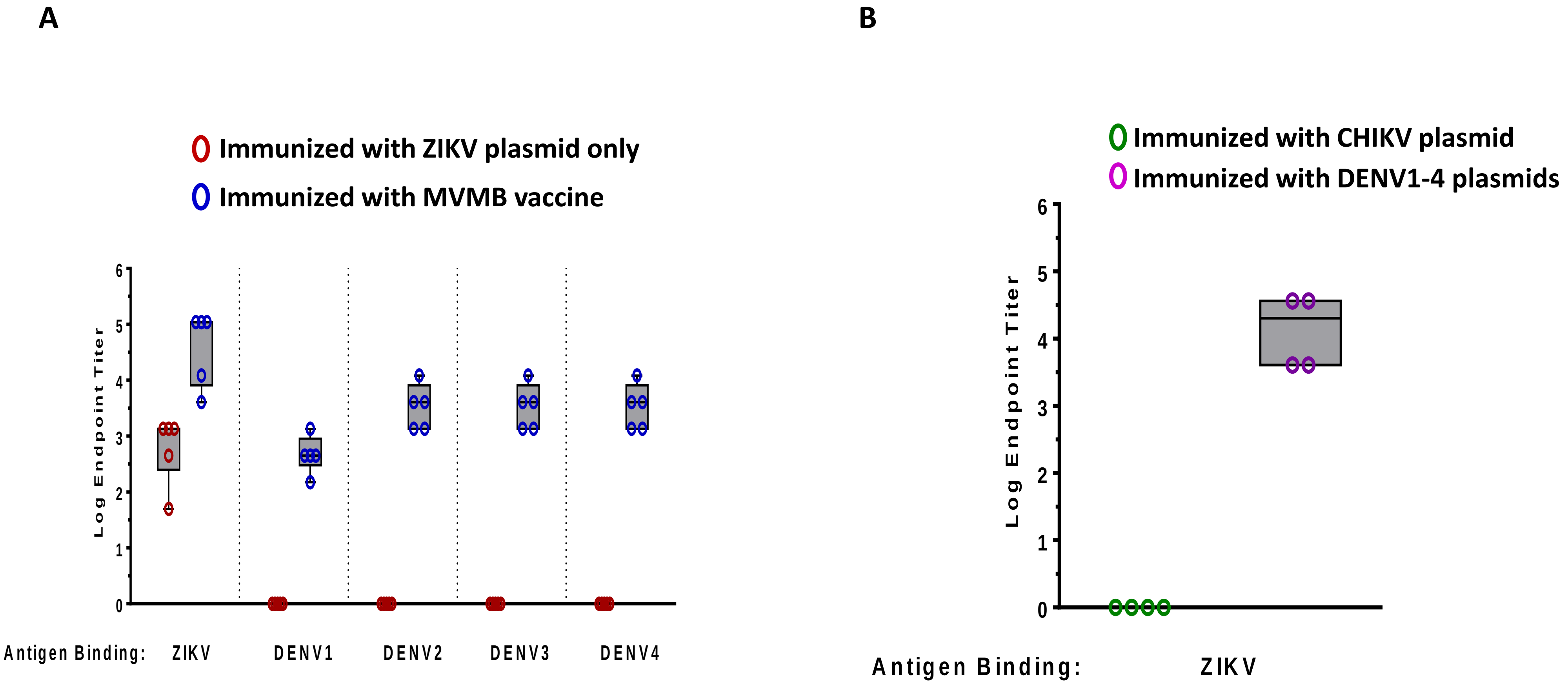

Supplement Figure S3. Cross reactive humoral responses between DENV1-4, ZIKV and CHIKV vaccines in guinea pigs. Guinea pigs were immunized with MVMB or ZIKV plasmids only (A); DENV1-4 or CHIKV plasmids (B) to test cross reactivity at week 9. Increased ZIKV antibody binding detected with MVMB immunization over ZIKV immunization alone (A). Antibodies elicited by DENV immunization could bind to ZIKV antigen; antibodies elicited by CHIKV immunization does not bind to ZIKV antigen (B).

Supplemental Material and Methods

S1 *Cross reactive antibody responses and plasmids interference among EBOV, MARV and LASV DNA vaccines in guinea pigs*

Guinea pig immunizations: For MHFV vaccine studies, guinea pigs were injected intradermally by Mantoux method on the flank followed by CELLECTRA® ID-EP on days 0, 21, and 42 for a total of three immunizations at a dose of 0.1 mg in 0.1 mL each of pEBOV, pMARV, and pLASV plasmids for total 0.3 mg pDNA. Immunizations were delivered as individual plasmids at 1 treatment site, or a two-plasmid cocktail at 1 treatment site, or a two-plasmid cocktail with an additional third individual plasmid given at 2 treatment sites, or a three-plasmid cocktail given at 2 treatment sites. In total 8 groups were utilized. Sera were collected on day 42 for ELISAs.

S2: *Cross reactive cellular responses between DENV1-4, ZIKV and CHIKV vaccines in mice*

Mouse immunizations: For cross reactive cellular responses studies, mice were injected intramuscularly followed by CELLECTRA® IM-EP on days 0, 14, and 28 for a total of three immunizations with either a dose of 25 μg pZIKV, or a cocktail dose of 100 μg of mixed pDENV1-4 plasmids (25 μg each plasmid), or a 25 μg pCHIKV, or a 25 μg pVAX (control) over 1 treatment sites per animal. Splenocytes on day 35 for IFNγ ELISpot analyses.

Enzyme-linked immunospot (ELISpot) assays

To assess cellular IFNγ responses, mouse interferon (IFN)γ ELISpot assays were performed using commercial Mabtech IFNy ELISpot kits (Mabtech, Sweden). Briefly, 96-well ELISpot plates pre-coated with capture antibody were blocked with R10 medium overnight at 4°C. The following day, 200,000 mouse splenocytes PBMCs in R10 media were added to each well and incubated at 37°C in 5% CO2 in the presence of peptide pools consisting of 15-mers overlapping by 9 amino acids and spanning the length of ZIKV-prME, CHIKV E1, E2, E3, or DENV1, 2, 3, 4-prME proteins (for MMBV); DMSO (negative control); and ConA (positive control for mouse). After 18-20 hours, plates were washed and developed according to the manufacturer's protocols, and IFNγ positive spots were counted using an automated ELISpot reader (CTL, Shaker Heights, OH). Antigen-specific responses were determined by subtracting the number of spots in DMSO-treated samples from peptide-treated wells. Results are shown for individual animal spot-forming units (SFU)/106 PBMCs obtained for triplicate wells.

S3 *Cross reactive humoral responses between DENV1-4, ZIKV and CHIKV vaccines in guinea pigs.*

Guinea pig immunizations: For MMBV vaccine studies, guinea pigs were injected intradermally by Mantoux method on the flank followed by CELLECTRA® ID-EP on days 0, 21, and 42 for a total of three immunizations with a cocktail of 0.1 mg in 0.1 mL each of pZIKV, pDENV1-4, and pCHIKV plasmids for total 0.6 mg pDNA spread across 6 treatment sites per animal per treatment or 0.1 mg in 0.1 mL of pZIKV. For CHIKV and DENV1-4 vaccine studies, guinea pigs were injected intradermally by Mantoux method on the flank followed by CELLECTRA® ID-EP on days 0, 21, and 42 for a total of three immunizations with a cocktail of 0.1 mg in 0.1 mL each of pDENV1-4 for a total of 0.4 mg or pCHIKV plasmid in 1 treatment site per animal per treatment. Sera were collected on days 0, 21, 42, and 63 for ELISAs.

Is this the right spot for this?
